# Alterations in resting-state functional connectivity of the default mode network in amnestic mild cognitive impairment: an fMRI study

**DOI:** 10.1186/s12880-017-0221-9

**Published:** 2017-08-16

**Authors:** Moyi Li, Guohua Zheng, Yuhui Zheng, Zhenyu Xiong, Rui Xia, Wenji Zhou, Qin Wang, Shengxiang Liang, Jing Tao, Lidian Chen

**Affiliations:** 1 Department of Rehabilitation, The First Affiliated Hospital of Nanchang University, Nanchang, 330006 China; 2 College of Health Information Technology and Management, Shanghai University of Medicine & Health Sciences, Shanghai, 201318 China; 3 College of Rehabilitation Medicine, Fujian University of Traditional Chinese Medicine, Fuzhou, 350122 China; 4 The Second Affiliated Hospital of Shandong University of Traditional Chinese Medicine, Jinan, 250001 China; 5 Physical Science and Technology College, Zhengzhou University, Zhengzhou, 450001 China; 6 Division of Nuclear Technology and Applications, Institute of High Energy Physics, Chinese Academy of Sciences, Beijing, 100049 China; 7 Beijing Engineering Research Center of Radiographic Techniques and Equipment, Beijing, 100049 China; 8 Fujian Key Laboratory of Rehabilitation Technology, Fuzhou, 350003 China

**Keywords:** Amnestic mild cognitive impairment, Default mode network, Functional magnetic resonance imaging, Functional connectivity, Independent component analysis

## Abstract

**Background:**

Amnestic mild cognitive impairment (aMCI) is characterized by cognitive functional decline, especially in memory. Resting-state functional magnetic resonance imaging (fMRI) has been widely used in neuroimaging studies that explore alterations between patients and normal individuals to elucidate the pathological mechanisms of different diseases. The current study was performed to investigate alterations in the functional connectivity of the default mode network (DMN) in aMCI patients compared to healthy elderly controls, as well as further define the association between neurological alterations and memory function.

**Methods:**

Twenty-five aMCI patients and 25 healthy individuals were recruited and underwent both fMRI and neuropsychological examinations. fMRI data was analyzed by independent component analysis.

**Results:**

Compared to healthy individuals, aMCI patients exhibited a significant increase in functional connectivity between the DMN and right-middle and right-superior frontal gyri, left-middle occipital gyrus, and left-middle temporal gyrus, but reduced functional connectivity between the DMN and left-middle and left-inferior frontal gyri and left insula. These alterations were found to be associated with reduced memory function.

**Conclusions:**

aMCI patients exhibited abnormal functional connectivity between the DMN and certain brain regions which is associated with changes in memory function associated with aMCI.

**Electronic supplementary material:**

The online version of this article (doi:10.1186/s12880-017-0221-9) contains supplementary material, which is available to authorized users.

## Background

Current evidence suggests that approximately 44 million individuals suffer from dementia or Alzheimer disease (AD) worldwide, and this figure is expected to more than triple by 2050 due to a rapidly expanding older adult population [1]. AD is a neurodegenerative disorder characterized by progressive dementia with widespread cognitive decline [2]. Mild cognitive impairment (MCI) is considered to be an intermediate stage between normal aging and AD [3, 4]. MCI patients show decline in cognitive functiongreater than that expected for one’s age and education level, but the severity is not enough to be classified as dementia; however, these individuals have a higher risk of developing dementia or AD than their age-matched, normal controls (10–15% annually for MCI versus 1–4% for controls) [5–7]. Moreover, the subtype of MCIwith memory complaint and deficit, namely amnestic MCI (aMCI), is consistently shown to have a higher risk of dementia than those without memory impairment [6]. More and more attention has been paid to preventative therapies and pathological processes associated with each stage of MCI in recent years because clinical trials of treatments for AD have failed. The growing consensus is that the target population for more effectively interventions is not individuals with diagnosed dementia but rather those with aMCI [8, 9].

Recently, resting-state functional magnetic resonance imaging (fMRI) has been increasingly utilized for studying the pathogenesis of MCI. Many researchers have begun to explore alterations within the resting-state brain network which may directly relate to disease pathology. The idea of the default mode network (DMN) has attracted many neuroimaging experts to examine alterations in the resting-state brain physiology of normal aging subjects and those with neurological disorders predominantly via fMRI [10]. The DMN is believed to anatomically involve the precuneus (PCu)/posterior cingulate cortex (PCC), medial prefrontal cortex (MPFC), medial temporal lobe, including the hippocampus and adjacent cortex, and inferior parietal lobule, as well as temporoparietal junction,retrosplenial cortex and the lateral temporal cortex, which play vital roles in cognition and memory [11–13]. Many studies have demonstrated that breakdown of DMN connectivity may underlie cognitive function loss in MCI subjects. For example, the intrinsic functional connectivity in the hippocampus, parahippocampal gyrus, PCu [14], PCC, inferior parietal lobule [15], and medial temporal and/or bilateral medial frontal lobes [16] has been shown to be decreased in MCI subjects compared with healthy controls. The functional connectivity between different regions within the DMN, such as between the hippocampus and PCC [17, 18], PCC and MPFC, and/or PCC and PCu [19], has also been reported to be absent or decreased in MCI patients. Hence, such neuroimaging findings could be considered as potential biomarkers of MCI. However, most previous results were obtained based on region-of-interest analyses, making it difficult for researchers to come to a unanimous conclusion considering the limitations of this approach with the relative arbitrariness of the region-of-interest [20].

In the present study, we investigated the presence of DMN alterations in aMCI patients compared with healthy, elderly controls using independent component analysis (ICA). In particular, we examined the relationship between network functional connectivity and different brain regions in aMCI patients to better clarify the neuropathological mechanism associated with aMCI. Furthermore, we assessed whether the changes in brain network functional connectivity correlated with severity of global cognitive performance.

## Methods

### Participants

Twenty-five participants diagnosed with aMCI according to Peterson diagnostic criteria were recruited from the Cangxia and Fengdanbailu communities in Fuzhou City (China) [21]. All patients were males or females aged 60 years or older, had recent subjective memory complaint, showed objective memory decline defined by a Wechsler Memory Scale-Chinese Revision (WMS-CR) score less than 1.5standard deviations below normative means, normal or near-normal general cognitive function defined by a Mini-Mental State Examination (MMSE) score less than normality with adjustment for age and education, a preserved activities of daily living (ADL) scale score less than 18, and absence of dementia. Another 25 healthy, elderly control subjects without memory complaint were also recruited. All aMCI and control participants were right-handed and were without MRI contraindications.

### fMRI data acquisition

Images were collected using a 3.0-Tesla General Electric scanner (Milwaukee, WI, USA) with an eight-channel phased-array head coil. All subjects were instructed to lie still with their eyes closed without falling asleep, stay relaxed, and not think of anything in particular. Resting-state scans were acquired using an echo planar imaging sequence with the following parameters: time resolution = 2100 ms, echo time = 30 ms, field of view = 200 × 200 mm, flip angle = 90°, slice thickness = 3 mm with a 0.6-mm gap, 42 slices, 64 × 64 matrix, and phases per location = 160. T1 three-dimensional magnetization-prepared rapid gradient-echo imaging was acquired in the same session with the following parameters: echo time = min, field of view = 240 × 240 mm, flip angle = 15°, inversion time = 450 ms, slice thickness = 1 mm, and 164 slices per acquisition). Both behavioral examinations and fMRI scans were completed within a week after enrollment.

### fMRI data preprocessing

fMRI data preprocessing was performed using the Oxford Centre for Functional MRI of the Brain’s (FMRIB) Software Library (FSL version 5.0.8, Oxford, UK; http://www.fmrib.ox.ac.uk/fsl). Nine-parameter nuisance signals, including global, white matter, and cerebral spinal fluid signals and six head motion parameters, were extracted and removed using the Brain Extraction Tool in FMRIB, and a mean functional image was created for each subject. Next, functional images were spatially smoothed with a 6-mm full-width at half-maximum Gaussian smoothing kernel in FMRIB. Data were then band-pass filtered at 0.01 to 0.10 Hz to reduce the influence of low frequency noise and effects of higher frequencies, such as respiratory and cardiac signals. In addition, each participant’s functional images were coregistered to their corresponding skull-stripped anatomical image and then registered to the Montreal Neurological Institute 152 stereotactic template using linear affine transformations with 12 degrees of freedom.

### fMRI data analysis

Analysis of fMRI data was performed using multivariate exploratory linear optimized decomposition into independent components [22]. Probabilistic ICA was applied to derive each group’s (*n* = 50 total) resting-state network activity at 20 components. The DMN was identified according to functional networks described in earlier studies, and the similarity between our data and the template networks derived from 1414 participants was calculated [23].

Then, a dual-regression technique was applied [23]. First, the priori defined DMN was used as a spatial regressor in a general linear model (GLM) to extract each subject’s temporal dynamics. These time-courses were then used as a set of temporal regressors in our GLM to generate subject-specific maps associated with the different group-level independent components. Finally, group analysis was performed with the whole brain and subject-specific maps from the second GLM, which represented the functional connectivity strength of each voxel with the DMN.

To explore the relationship between changes in functional connectivity and cognitive behavior, a regression analysis was performed between subject-specific network maps and each subject’s WMS-CR memory quotient (MQ). A threshold of voxel-wise Z > 2.3 and cluster-level family wise error (FWE) correction for multiple comparison corrections of *P* < 0.05were used. Moreover, age, gender, and education level were considered as covariates in this GLM.

### Statistical analysis

Statistical analyses were performed with SPSS version 18.0 software (SPSS Inc., Chicago, IL, USA) for demographic and neuropsychological data, as well as correlation analyses. The normality of continuous variables was examined using the Shapiro-Wilk test. Normally distributed data were expressed as means ± standard deviations and analyzed by Student’s t-test. Non-normally distributed data were analyzed using the nonparametric Mann-Whitney U-test and reported as the median and interquartile range. A Chi-square test was used to assess gender differences between the two groups. The mean Z-values of DMN regions with significant differences between the two groups were extracted, and correlations with neuropsychological scores were analyzed using a Spearman correlation analysis or Pearson correlation analysis if data had non-normal distribution. A *P* < 0.05 was considered to be significantly different.

## Results

### Demographic and neuropsychological data

Demographic and neuropsychological characteristics of both groups are described in Table 1. Gender, age, and MMSE and ADL scores were not significantly different between aMCI and control groups (all *P* > 0.05). Interestingly, the education level of aMCI patients was significantly higher than for healthy controls (*P* = 0.012), but their memory ability measured by WMS-CR was significantly lower than the healthy control group (*P* < 0.001).

**Table 1:** Participant demographics and baseline neuropsychological test scores

	MCI (*N* = 25)	HC (*N* = 25)	*P* value
Gender (M/F)	9/16	11/14	0.564
Age (years): Mean ± SD	64.56 ± 4.984	62.84 ± 2.794	0.219
Education (years): Mean ± SD	11.44 ± 2.931	9.32 ± 2.495	0.012
MMSE: Mean ± SD	26.88 ± 1.856	27.60 ± 1.732	0.154
ADL	14	14	1
WMS-CR MQ	91.28 ± 11.07	110 ± 8.902	<0.001

*ADL* activities of daily living, *HC* healthy control, *MCI* mild cognitive impairment, *MMSE* Mini-Mental State Examination, *WMS-CR MQ* Wechsler Memory Scale-Chinese Revisionmemory quotient, *SD* standard deviation

### Differences in DMN functional connectivity

**Fig. 1:**
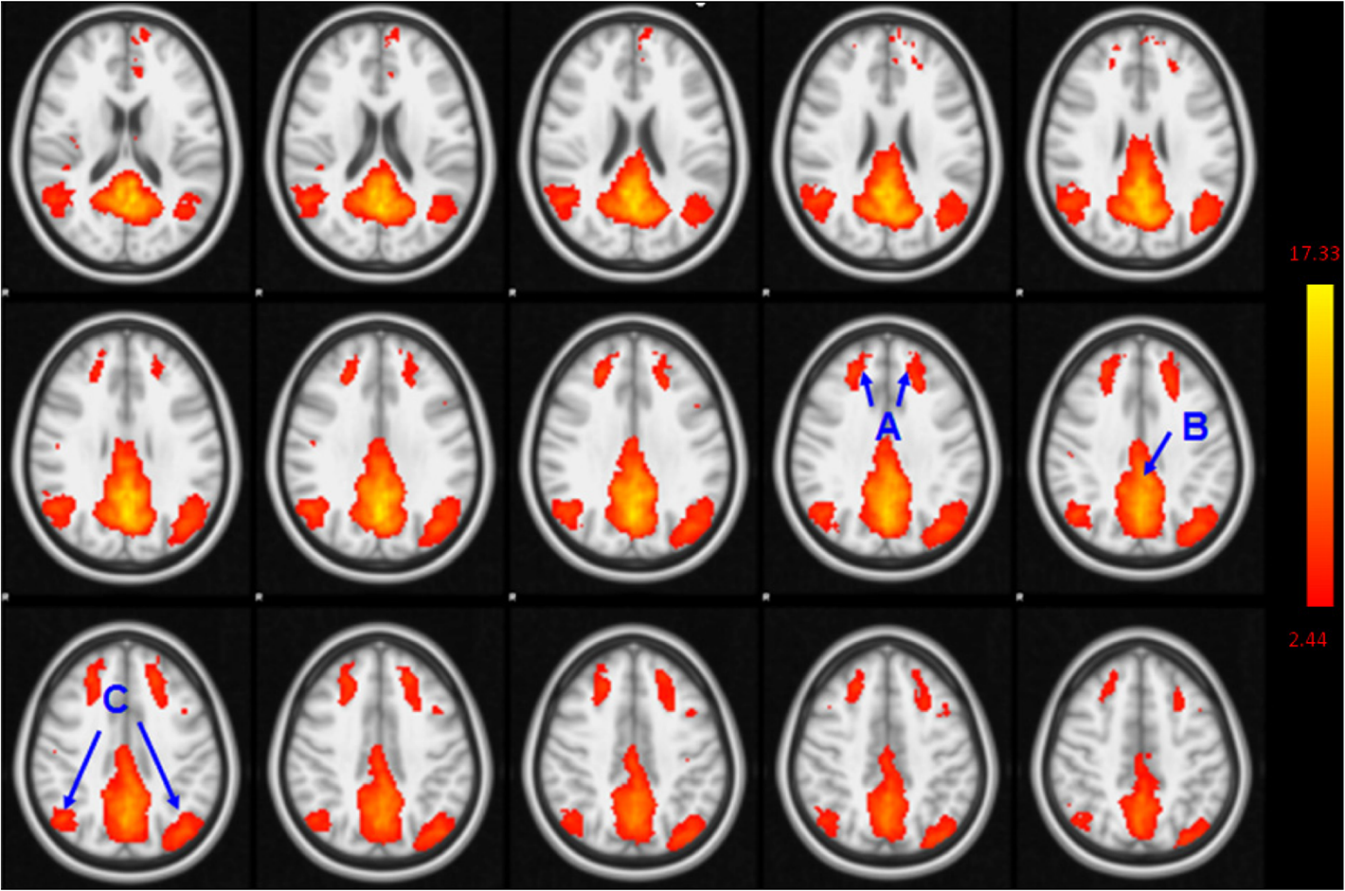
The DMNof all participants derived from ICA. Brain regions observed in the network included the bilateral medial prefrontal (**a**), inferior parietal lobe/angular gyrus (**b**) and PCu/PCC (**c**)

The DMN obtained from ICA was in conformity with previously published results [23], which included the MPFC, anterior cingulate cortex, parietal cortex, and Pcu/PCC (Fig. 1). The correlation coefficent (r-value) between the independent component we chose and the template network was 0.56. All valid resting-state components derived from ICA are shown in Additional file 1: Table S1.

**Fig. 2:**
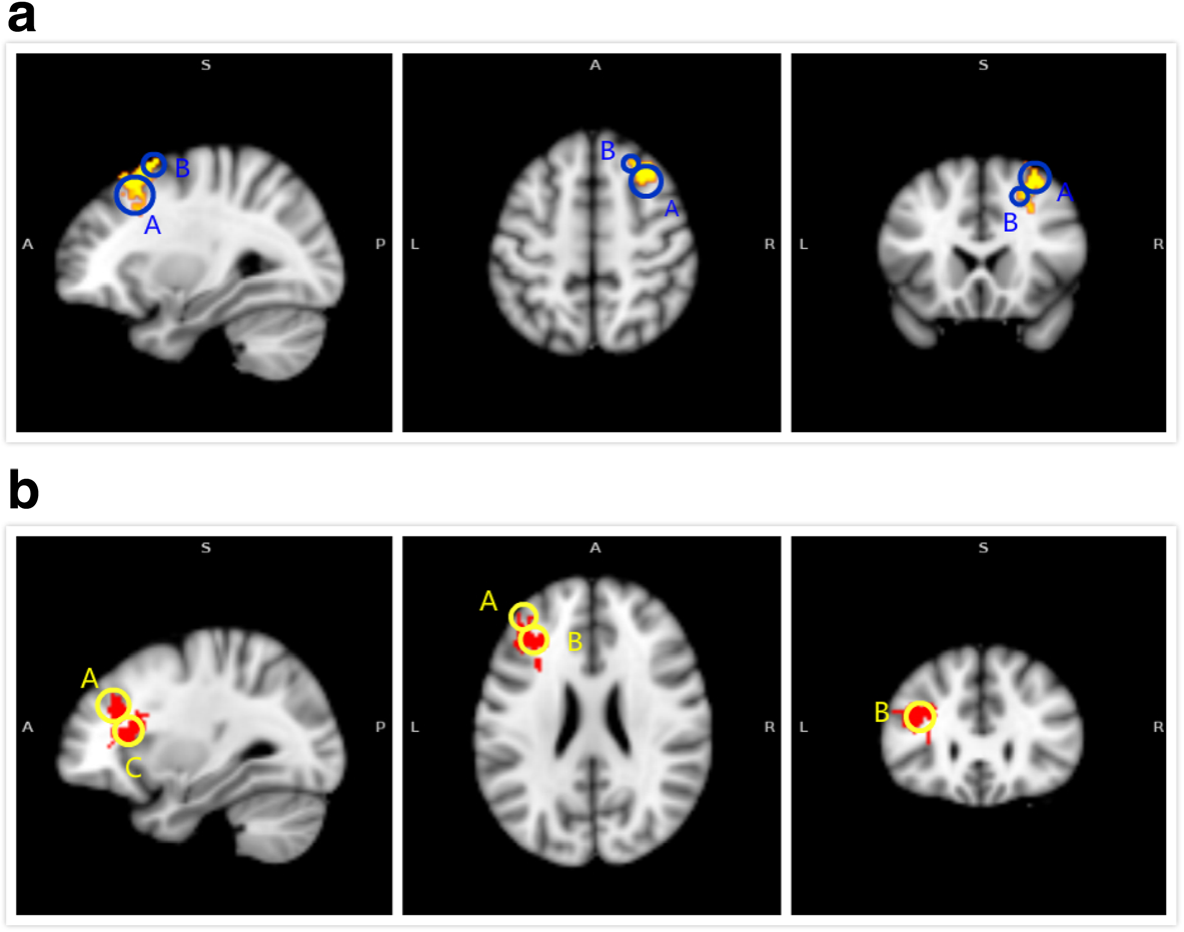
Abnormal functional connectivity of the DMN in MCI patients compared to healthy controls. **a** The yellow color indicates brain regions exhibiting increased functional connectivity (A: right-middle frontal gyrus; B: right-superior frontal gyrus, left-middle occipital gyrus, and left-middle temporal gyrus) in MCI patients compared with healthy controls. **b** The red color indicates brain regions exhibiting decreased functional connectivity (A: left-middle frontal gyrus; B: left-inferior frontal gyrus; C: left insula) in MCI patients compared to healthy controls

Compared with healthy controls, aMCI subjects exhibited a significant increase in functional connectivity between the DMN and right-middle and right-superior frontal gyri, left-middle occipital gyrus, and left-middle temporal gyrus. On the other hand, reduced functional connectivity was found between the DMN and the left-middle and left-inferior frontal gyri and left insula (Fig. 2a-b; Table 2).

**Table 2:** Comparison of significant differences in functional connectivity between MCI and HC group

Contrast	Brain regions	MNI coordinates (mm)			Peak Z-score	Cluster size (mm^3^)
		x	y	z		
MCI>HC	R. Middle Frontal Gyrus/ R. Superior Frontal Gyrus	32	22	52	4.28	429
	L. Middle Occipital Gyrus/ L. Middle Temporal Gyrus	−50	−80	20	3.90	162
HC>MCI	L. Middle Frontal Gyrus/ L. Inferior Frontal Gyrus/ *L. insula*	−30	20	22	4.33	432

Abbreviations:*HC* healthy control, *L* left, *MCI* mild cognitive impairment, *MNI* Montreal Neurological Institute, *R* right

### Correlations between DMN functional connectivity and WMS-CR MQ

**Fig. 3:**
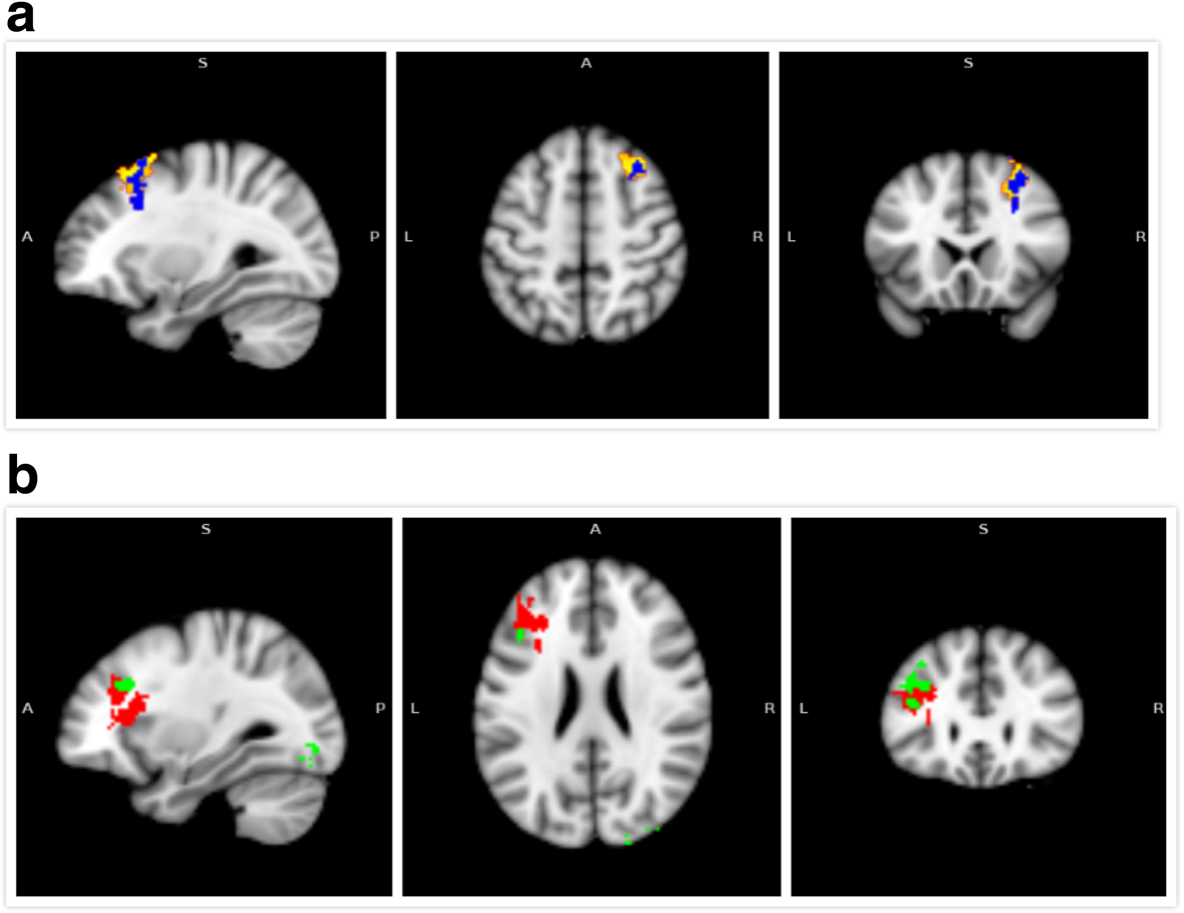
Correlation between functional connectivity alterations in the DMN and WMS-CR MQ changes across all participants. **a** The yellow color indicates brain regions exhibiting increased functional connectivity in MCI versus control subjects; the blue color indicates brain regions exhibiting a negative association between their DMN functional connectivity alterations with corresponding WMS-CR MQs across all participants. **b** The red color indicates brain regions exhibiting decreased functional connectivity in MCI patients compared to healthy controls; the green color indicates brain regions exhibiting a positive association between their DMN functional connectivity alterations with corresponding WMS-CR MQs across all participants

**Fig. 4:**
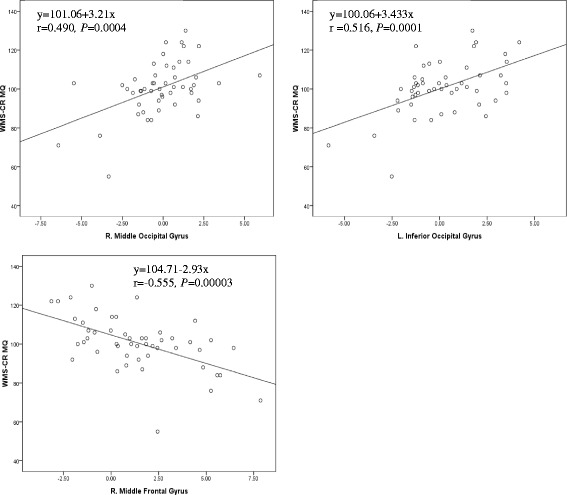
Correlation between functional connectivity alterations in the right-middle and left-inferior occipital gyri and right-middle frontal gyrus with WMS-CR MQ changes based on Pearson correlation analysis

Regression analysis between the DMN and WMS-CR MQ showed a negative association between the DMN and the left-middle frontal gyrus, right-middle and left-inferior occipital gyri, and left-middle temporal gyrus. A positive association was found between the DMN and right-middle and right-superior frontal gyri and right cingulate gyrus (Fig. 3a-b; Table 3). Pearson correlation analysis showed that WMS-CR MQ changes were positively correlated with functional connectivity alterations in the right-middle occipital gyri (*r* = 0.490, *P* = 0.0004) and left-inferior occipital gyri (*r* = 0.516, *P* = 0.0001),where as negatively correlated with functional connectivity alteration in the right-middle frontal gyrus (*r* = −0.555, *P* = 0.0003) (Fig. 4).

**Table 3:** Brain regions showing significant associations between WMS-CR MQsand corresponding functional connectivity

Contrast	Brain regions	MNI coordinates (mm)			Peak Z-score	Cluster size (mm^3^)
		x	y	z		
MCI>HC	R. Middle Occipital Gyrus/ *R. inferior* Occipital Gyrus/ R. Superior Occipital Gyrus/ R. Cuneus	36	−78	−6	4.01	443
	L. Middle Frontal Gyrus/ L. Superior Frontal Gyrus/ L. Inferior Frontal Gyrus/ L. Insula	−42	24	22	3.46	237
	L. Inferior Occipital Gyrus/ L. Middle Occipital Gyrus/ L. Fusiform	−34	−74	−12	4.69	224
	L. Middle Temporal Gyrus/ L. Inferior Temporal Gyrus	−52	−24	−14	3.65	154
HC>MCI	R. Middle Frontal Gyrus/ R. Superior Frontal Gyrus/ R. Cingulate Gyrus	28	16	32	4.55	228

Abbreviations:*HC* healthy control, *L* left, *MCI* mild cognitive impairment, *MNI* Montreal Neurological Institute, *R* right, *WMS-CR MQs* Wechsler Memory Scale-Chinese Revision memory quotients

## Discussion

Several previous studies have compared differences in the DMN between MCI and healthy subjects [14–19, 24, 25]. Most of them used region-of-interest (ROI) and task-fMRI to explore the functional connectivity or activation of regions within the DMN. However, results of previous studies varied widely due to different ROIs and tasks the researchers chose and therefore analysis of functional connectivity via resting-state fMRI requires a less complex task design and data-driven approach. Moreover, ICA can determine distinct components by capturing spatial independence and time-courses of resting-state data and thereby, reliably define different resting-state networks. In the current study, we examined functional connectivity within the DMN in a sample of aMCI patients and healthy controls using the ICA method and found an increase in functional connectivity between the DMN and right-middle and right-superior frontal gyri, left-middle occipital gyrus, and left-middle temporal gyrus, as well as decreased functional connectivity between the DMN and left-middle and inferior frontal gyri and left insula in aMCI patients compared to healthy controls. In addition, our findings also indicated these alterations in functional connectivity between the DMN and specified brain regions were closely associated with memory decline.

The frontal lobe is one of the most complicated brain regions and is involved in a variety of cognitive functions, especially memory [26, 27]. aMCI patients, characterized by memory deficits, often exhibit abnormal functional connectivity between the frontal lobe and other brain regions. As a result, the frontal lobe is considered to be a “hub” that is profoundly relevant to memory processing in MCI [28]. Sui et al. [29] reported that long-range functional connectivity density in the superior and middle frontal gyri was increased in MCI patients compared to healthy controls and also correlated with cognitive performance, allowing differentiation of MCI brain from controls. Zhao et al. [30] found that aMCI patients showed increased amplitude of low-frequency fluctuation (ALFF) signals in the left-superior and middle-frontal gyri compared with normal subjects. Similarly, our results showed that aMCI patients showed a significant increase in functional connectivity between the DMN and right-middle and right-superior frontal gyri.

The middle occipital gyrus is an important part of the primary visual cortex considered to play a role in the processing of visual recognition. Its abnormal connectivity might lead to impaired visual cognition in aMCI patients [31]. Cai et al. [32] reported that aMCI patients exhibited a significant increase in ALFF in the left-middle occipital gyrus and increased functional connectivity between the left-middle occipital gyrus and other regions. Golby et al. [33] also found that AD patients exhibited deficient explicit memory but had normal implicit memory, which was encoded in the occipital cortex. In the current study, results revealed an increase in functional connectivity between the DMN and left-middle occipital gyrus in aMCI patients compared with healthy controls, which might be helpful for maintaining normal implicit memory in patients with aMCI. In addition, current results also demonstrated a significant decrease in functional connectivity between the DMN and left-inferior frontal gyrus in MCI patients, which is consistent with a prior study [34]. This is likely because encoding and retrieval of memory in healthy older adults is primarily driven by the hippocampus and inferior frontal gyrus [35]. Thus, the decreased functional connectivity of aMCI patients may result in problems with encoding and retrieval of memory.

Some possible limitations to the current study may exist. First, our sample size was relatively small (*n* = 25 per group), which might have affected the statistical power of results and might induce sampling biases. Second, considering the difficulty associated with recruitment, we did not use a matched-pair study design. This likely led to the significant difference in education level between the two groups and may have induced bias regarding cognitive measures between groups. Additionally, it is difficult to derive causal associations between changes in functional connectivity and cognitive ability due to the cross-sectional design of our study. Further studies with larger sample sizes are needed to strengthen the results, and might apply a matched-pair design with a control group that is age-, gender- and education-level- matched to reduce bias. Moreover, longitudinal studies with follow-up periods are needed to investigate whether functional connectivity alterations of the DMN in aMCI could be an early biomarker for higher risk of conversion to dementia in the future.

## Conclusions

In summary, the present study was conducted to explore the alterations of the resting-state functional connectivity of the DMN in patients with MCI. The results demonstrated that MCI exhibited significantly increased functional connectivity between the DMN and right-middle and right-superior frontal gyri, left-middle occipital gyrus, and left-middle temporal gyrus, but reduced functional connectivity between the DMN and the left-middle and left-inferior frontal gyri and left insula. These alterations were associated with memory changes in MCI patients, which suggested that the altered DMN functional connectivity might be useful for the preclinical identification of dementia.

## Additional file

Additional file 1: Table S1. Valid resting-state components derived from ICA. (DOCX 13 kb)

## Abbreviations

AD: Alzheimer disease
ADL: Activities of daily living
ALFF: Amplitude of low-frequency fluctuation
aMCI: Amnestic mild cognitive impairment
DMN: Default mode network
fMRI: Functional magnetic resonance imaging
FMRIB: Functional MRI of the brain’s
FSL: Functional MRI of the brain’s software library
GLM: General linear model
ICA: Independent component analysis
MMSE: Mini-mental state examination
MPFC: Medial prefrontal cortex
MQ: Memory quotient
PCC: Posterior cingulate cortex
PCu: Precuneus
WMS-CR: Wechsler memory scale-chinese revision
