# Supplementary material for: Alterations in resting-state functional connectivity of the default mode network in amnestic mild cognitive impairment: an fMRI study
Source: BMC Med Imaging. 2017 Aug 16;17:48. doi: 10.1186/s12880-017-0221-9 (PMC5559812; doi:10.1186/s12880-017-0221-9)
Supplement: Additional file 1: Table S1. — Valid resting-state components derived from ICA. (DOCX 13 kb) [file 12880_2017_221_MOESM1_ESM.docx]

**Table S1. Valid resting-state components derived from ICA**

| No. of component | Name of component |
| --- | --- |
| 1 | visual network |
| 2 | right frontoparietal network |
| 3 | left frontoparietal network |
| 4 | default mode network |
| 6 | right somatomotor network |
| 7 | dorsal attention network |
| 8 | auditory network |
| 9 | cerebellum network |
| 10 | left somatomotor network |
| 11 | executive control network |
| 12 | salience network |
| 13 | ventral attention network |
| 14 | sensorimotor network |
